# JaPaFi: A Novel Program for the Identification of Highly Conserved DNA Sequences

**DOI:** 10.3390/v2091867

**Published:** 2010-08-31

**Authors:** Aliya Sadeque, Marina Barsky, Francesco Marass, Peter Kruczkiewicz, Chris Upton

**Affiliations:** 1 Department of Biochemistry and Microbiology, University of Victoria, Victoria, BC, V8W 3P6, Canada; E-Mails: aliya.sadeque@gmail.com (A.S.); fmarass@uvic.ca (F.M.); peterk@uvic.ca (P.K.); 2 Department of Computer Science, University of Victoria, Victoria, BC, V8W 3P6, Canada; E-Mail: marina.barsky@gmail.com

**Keywords:** Poxvirus, bioinformatics, highly conserved sequences, approximate match, conserved function, JaPaFi

## Abstract

We describe the use of Java Pattern Finder (JaPaFi) to identify short (<100 nt) highly conserved sequences in a series of poxvirus genomes. The algorithm utilizes pattern matching to identify approximate matches appearing at least once in each member of a set of genomes; a key feature is that the genomes do not need to be aligned. The user simply specifies the genomes to search, minimum length of sequences to find and the maximum number of mismatches and indels allowed. Many of the most highly conserved segments contain poxvirus promoter elements.

## 1. Introduction

One of the fundamental principles of molecular evolution is that extensive sequence similarity implies homology and frequently, conservation of function [1]. In comparative genomics, the complete genomic information from one organism—including gene number, sequence, location, and length as well as features of the non-coding regions—is compared to that of another organism in order to gain insights about phylogeny and conserved functions [2]. The applications of methods in comparative genomics are broad, with recent applications including—amongst others—the identification of genes and regulatory elements [3,4], the functional characterization of genes [5,6], and prediction of protein structures [7,8].

In studies of non-coding regions, sequences that display particularly high degrees of conservation are regarded as good candidates for regulating gene expression [1,3]. This point is illustrated by the discovery of the Conserved Sequence Element (CSE) in 2003 during the genome sequencing of the Yaba Monkey Tumor Virus, a member of the Yatapoxvirus genus [9]. While sequencing the genome, a 42 nucleotide (nt) sequence was identified that seemed unusually well conserved; unusual in both its length and the fact that it was almost perfectly conserved between members of four different poxvirus genera. Although subsequent experiments on the CSE ultimately led to its classification as a promoter element in poxviruses [10], the CSE remains unusual because it is remarkably well conserved for a promoter, it is longer than the average poxvirus promoter (which is normally in the range of ∼30 nts) [11], and it actually contains both early and late promoter elements that are believed to act on an early/late gene [11,12]. The presence of both early and late promoter elements, as well as the very high conservation of the CSE, makes it more complex than other characterized promoters [10,13–14].

The Java Pattern Finder (JaPaFi) project arose from the need for a way to identify short (<100 nts), highly conserved sequences *de novo* (as opposed to searching for matches to a known sequence) without requiring an alignment of the DNA sequences being investigated. Classically, search tools such as BLAST have been used to search for sequence matches; however, since BLAST, PROSITE and other database search tools require input query sequences they are not appropriate for the identification of previously unknown motif-like matches. It is important to note that unlike BLAST, JaPaFi has no minimum *word* size that must be matched and indels may occur in part of the set of differences between the sequence patterns; thus JaPaFi can detect smaller patterns than a tool based on a BLAST-type algorithm. Also, with any alignment-based method, no matter how sophisticated the alignment algorithm, problems arise in the face of non-colinearity, as is the case with poxviruses. The tools that are currently available, such as WABA (Wobble Aware Bulk Aligner) [15], V-match (large-scale aligning) [16] and MGA (Multiple Genome Aligner) [17], are incapable of accounting for the rearrangement of large portions of the genome between related species [18]. Furthermore, among alignment-independent approaches, there remains a tradeoff between factors of value to scientists—namely speed, accuracy of results, and disk usage—with the paradigm being a program that will identify previously unknown sequence matches in a reasonable time frame and with reasonable disk usage. All of these factors continue to pose limitations to currently available tools. For instance, a motif-finding tool called Meme has been developed that identifies motifs of specified length within a set of sequences, however, it can only accept inputs of up to 60,000 characters total [19], which makes it impossible to run the program on a set of poxvirus genomes ranging from 150–350 kb.

Here we describe the JaPaFi tool, which utilizes a novel method to identify approximate matches in a set of genomes. The term *approximate match* refers to the fact that there are a user-specified number of positions that vary. We furthermore present an application of JaPaFi in which 11 highly conserved sequences have been identified in a set of seven poxvirus genomes. Preliminary bioinformatics analysis has been conducted on these sequences, which suggests most are likely to be associated with viral promoters.

## 2. Materials and Methods

### 2.1. Description of Java Pattern Finder (JaPaFi)

The main purpose of this tool is to find substring patterns that occur in N genomes. There are two parameters set by the user: minimum pattern length *S* and maximum number of differences *K*. The substring is considered interesting if it repeats in each of N genomes and has a length of at least *S*. The pattern substring can be found at least once in each genome, but need not be found as an exact replica, but instead as “almost exact” – *K* differences are allowed between an interesting pattern and the substring found in each of N genomes. Differences between the pattern and each substring mean that by applying no more than K edit operations (deletions, insertions, substitutions) to each substring we can convert it into the pattern. Thus, the program extracts similar (almost identical) substrings from a set of genomes (strings). It checks, in turn, all the substrings of the input strings, thus it is an extension of a well-known all–against–all string comparison problem introduced in [20]; each substring that occurs in all input strings with up to *K* differences, represents a new pattern group. After all such pattern substrings have been found, the program collects the start positions of the corresponding substrings related to a given pattern string by the similarity threshold. Given parameters *K* and *S*, the program finds all substrings, which can be converted into a pattern string by not more than *K* edit operations. This means that any other substring of length of at least *S* does not occur in all input strings with up to *K* differences.

In order to locate the approximate matches, the program performs two computations. In the first part, called Iterative Filtering, each pair of sequences is processed using the modification of the *APBT* algorithm described in [21]. The original *APBT* finds all approximate matches of length of at least *S* and with up to *K* differences in two strings. As an input, the modified algorithm takes two strings and two arrays of bits corresponding to all the start positions in these two input strings. The bit in the bit array is set to 1 if the approximate match can start at this position. When the input string is processed for the first time, all bits in its array are set to 1. After *APBT* finds all approximate matches bounded by *K* errors in a currently processed pair of input strings, the bits are set to 1 only at the start positions of these matches. These newly marked arrays are passed to the next iteration of the *APBT* algorithm, which will process only the pairs of positions where the potential matches can occur. The number of marked positions for each input string decreases with each new pairwise iteration.

In the second phase of the program, we collect all substrings of length *S* that start at the positions obtained as a result of the previous filtering step, remove duplicates, and, for each substring create a separate output group. Next, for each such reference substring, the positions of all approximate matches in all input strings are collected, such that each approximate match can be converted into the reference substring by no more than *K* edit operations. In this way, we find all the substrings of length *S* which are the most similar for all the input strings, since all the other substrings of length *S* would have more than *K* differences between them.

The algorithm is asymptotically quadratic in the length of each input string, so its scalability is limited to approximately 300,000 characters per input string. The overall efficiency heavily depends on the parameters *S* and *K*, which influence the number of start positions remaining after each step of Iterative Filtering. If the number of remaining positions (and therefore size of an output) is too big, the user can either decrease *K* or increase *S*. We were able to run Japafi with 12 poxvirus genomes ranging in size from approximately 150–300 kb; runtimes ranged from 30–90 mins for 0–3 differences. It should be noted that the program is intended to search relatively distantly related sequences for small (<100 nt) matches.

**Figure 1.**
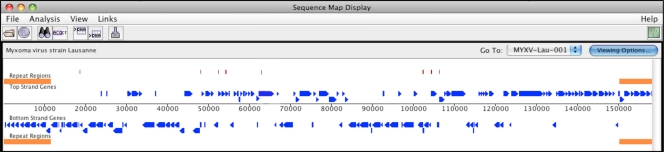
MYXV genome map with JaPaFi hits. Blue arrows are MYXV open reading frame (ORFs) and the red tick marks above are JaPaFi hits. Orange bars at the right and left extremities are the inverted terminal repeat regions of the genome.

JaPaFi can be used through a simple graphical interface in which users can upload any genome sequences in FASTA format from disk, or load specific genome sequences directly from the Virus Orthologous Clusters database, which is available through the Viral Bioinformatics Resource Center [22]. Users can enter *length* (*S*) and *allowed number of differences* (*K*) parameters into text entry fields and run JaPaFi. The output appears in a Results window and can be exported as simple-text to be saved to disk, or converted into a format that enables visualization of the hits for a particular genome against an annotated genome map using the available Viral Genome Organizer software [22] (Figure 1).

### 2.2. Genomes included in this study

**Figure 2.**
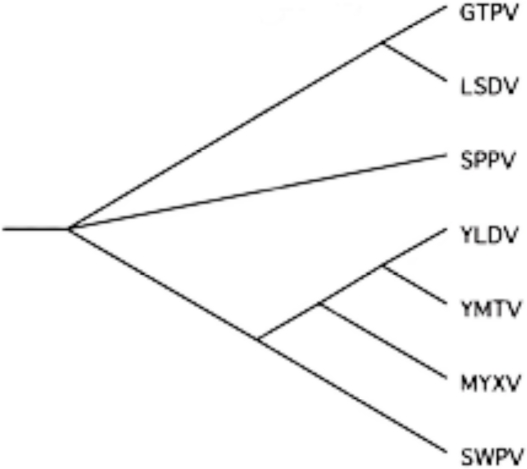
Cladogram made using a ClustalW whole genome alignment of the seven poxvirus genomes.

The set of seven genomes (Table 1) in which the CSE had been identified was selected in order to address the question of whether the CSE was in fact unusual in its size and degree of conservation or whether other comparable sequences were present within that set. All seven of these genomes were from the poxvirus subfamily *Chordopoxvirinae*. Any two genomes within this set of seven were between 56–98% identical. A cladogram of these genomes is shown in Figure 2.

## 3. Results and Discussion

### 3.1. Counting the number of hits as the parameters of length and number of differences are varied

**Figure 3.**
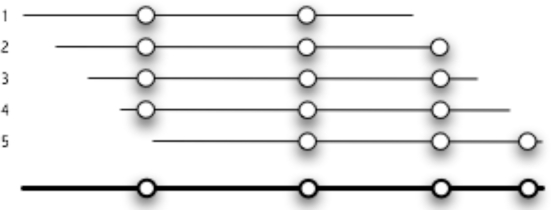
Fixed length patterns overlap to highlight longer regions of conservation.

JaPaFi was run for a series of different parameter combinations in order to observe the effects of altering length and allowed differences on the number of conserved sequences, or hits, identified. The nature of the JaPaFi output is such that the set of pattern matches identified for a particular parameter set includes some that overlap; if they overlap by 1 or more characters, these can be merged into larger contiguous regions (Figure 3). Most of the hits visualized are, therefore, an amalgamation of several pattern matches.

Hit counts were determined by visualizing the output of the program for each parameter combination against a genome map of the *Myxoma virus* genome, which served as our model genome, using the Viral Genome Organizer software. The numbers of hits for each parameter combination were recorded in a hit-count matrix (Table 2).

We chose a set of 11 hits for further examination based upon their consistent appearance in the sets of longest sequences for any given number of allowable differences (Table 3).

For each of the hits, a Logo [23] was created from the multiple alignment of the seven sequences using the WebLogo program [24]. The set of 11 hits consists of two hits from coding regions alone, and nine hits that contain, between them, promoter elements corresponding to 13 different genes (due to the fact that some of these were bidirectional promoters acting on two adjacent genes).

### 3.2. Quantifying the degree of conservation

A scoring method was established to quantify the degree of conservation of the hits based on the logos of the hits constructed using the Weblogo application. The heights of the nucleotides at each position of the Logo were extracted directly from the Weblogo program using an in-house script. *Position scores* were taken to be the height of the tallest (most frequently appearing) nucleotide at a given position in the sequence. Taking the height of only the most frequently appearing nucleotide into account in the score ensured that higher scores would be gained for more conserved positions, since the greatest height possible is seen at perfectly conserved positions. These position scores were then summated over the length of the sequence being examined to obtain *Total Information*.

This method provided a way to quantify the degree of conservation observed in these hits. To show that the hits were more conserved than would be expected, a set of control sequences was selected to establish a baseline for the expected level of conservation. A series of known poxvirus promoters were selected to serve as these controls since most of our hits contain promoters. Scores for the hits were compared to scores for the control sequences, and the hits were shown by a student’s t-test to score higher than control sequences, with p-values of 1 × 10^−3^ or less (Table 4).

This analysis indicates that the hits identified are significantly more conserved than expected for a region of comparable length containing a poxvirus promoter, according to this scoring method.

### 3.3. Identifying promoter elements within the hits

**Figure 4.**
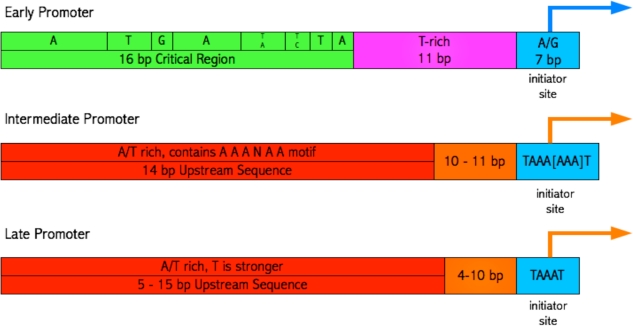
Known consensus of conserved poxvirus promoter elements.

Visualization of the sets of JaPaFi results for various parameter combinations against genome maps indicated that most of the hits mapped to regions overlapping and immediately upstream of the transcription start sites (promoter elements) of poxvirus genes. Poxvirus genes are temporally regulated at the transcription level by means of three classes of structurally distinct promoters, active at early, intermediate and late stages of the viral life cycle (Figure 4) [25]. To verify this observation, known motifs associated with the three temporal classes of poxvirus promoters were identified in logos of the hits in order to delineate putative promoter elements and visualize whether or not the hits are longer than expected once promoter elements are accounted for [13–14, 26–27].

**Figure 5.**
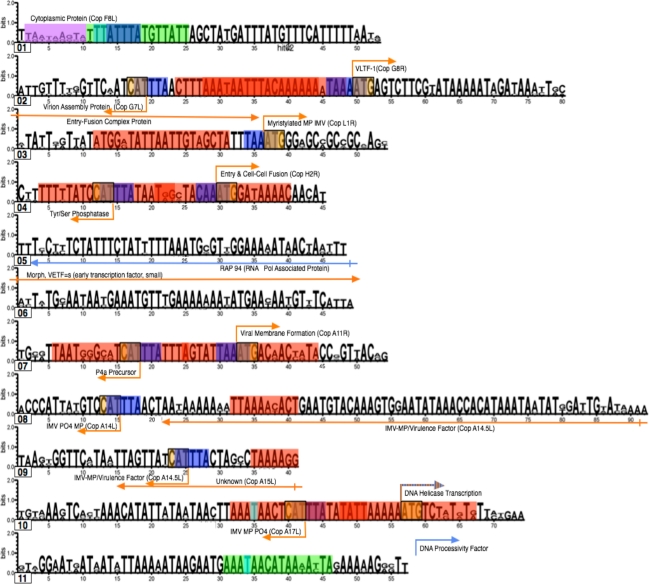
Annotated hit logos showing promoter elements; the hit number is indicated underneath (left) each logo. Blue arrows represent early genes, orange arrows represent late genes, and blue-and-orange striped arrows represent genes that are transcribed both early and late in the poxvirus life cycle. Highlighted promoter elements follow the color key shown in the diagram of the known consensuses of promoters (Figure 4).

In some cases, such as in Hit04, back-to-back promoters accounted for almost the whole length of the hit, while in others, lengthy stretches of highly conserved sequence flank the promoter elements (Figure 5).

### 3.4. Searching for short motifs shared between the hits and early, intermediate and late promoters

**Figure 6.**
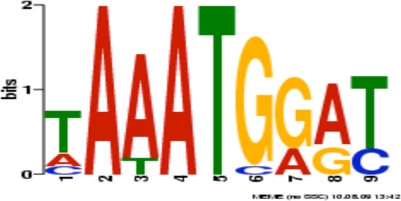
Logo of highest-scoring motif in hits and late gene upstream regions. E-value of 6.8 × 10^−1^ and 15 occurrences in four upstream regions and six different hits.

All hits were searched for smaller recurring motifs within them, in the 3–8 nt range, using MEME/MAST motif finder [28]. For each motif identified, MEME calculates an *E*-value that scores the overall match between the motif and all instances of this motif in the query sequences [19]. These *E*-values are used to judge the significance of the motifs by comparing them to a user-specified threshold *E*-value [19]. In this study, using the NCBI’s default threshold *E*-value as guidance, a threshold *E*-value of 10 was used [29]. MEME searches were conducted to identify 2–8 nt motifs shared between the hits and each of the three types of promoters in turn, hypothesizing that the program would pick up promoter elements appearing in the 30 nts upstream of the translation start site in the upstream sequences. One such motif appeared numerous times in both the hits and the promoters, appearing in 15 locations across six hits and four late gene upstream sequences (sometimes with multiple occurrences in a single hit or promoter if the hit contained more than one translation start site or if the promoter overlapped with another promoter). Its occurrences in upstream regions align at the translation start site and most of its occurrences in the hits coincide with actual translation start sites. With an e-value of 6.8 × 10^−1^, it is the only motif identified that can be considered statistically significant by virtue of being within two orders of magnitude of NCBI’s default threshold e-value of 10 (Figure 6).

This motif is similar to the Kozak sequence, which plays a major role in translation initiation in eukaryotic mRNA. The Kozak sequence is required for recognition of the initial AUG and functions by slowing down the speed of scanning by the ribosome [30]. It seems intuitive that late promoters contain a strong Kozak sequence because many of the late genes are translated into structural proteins, which are needed at high levels for building the progeny virus particles. A strong Kozak sequence in the context surrounding the initiator codon of a gene could modulate translation and enhance the amount of protein produced [31].

### 3.5 Coding Region Hits

#### 3.5.1. Conserved Protein Domains

Although nine out of 11 hits contained at least one promoter, two of the hits, Hit05 and Hit06, appeared only in coding regions and did not overlap with any known promoter regions or any other genes. Hit05 was found within the protein-coding region of the Viral Early Transcription Factor (VETF) gene, which encodes a promoter-binding protein with DNA-dependent ATPase activity that is involved in activating transcription of early genes in poxviruses [32]. The Hit05 sequence encodes the CNNEMFEKNMNNV region of the VETF protein. The EMBOSS PatMatMotif tool—a tool that searches the full PROSITE database of known protein motifs—was then used to query the PROSITE database for this amino acid sequence to see if it might be associated with a conserved protein domain or family, but no matches were found. The PROSITE database was then directly searched for matches against the amino acid sequence for the full VETF gene using the ScanProsite tool in order to see if it contained any known conserved protein domains, and if so, whether the hit region was a part of these conserved protein domains. This search returned matches to two conserved domains. One match was to a helicase domain, superfamilies 1 and 2, which binds ATP (PS51192). The other match was to the C-terminal helicase domain, superfamilies 1 and 2 (PS51194). These matches were distinct, non-overlapping regions in the protein sequence that were linked by a 145 nt sequence, and it was in this unmatched linker region that Hit05 was found, excluding it from the conserved domain matches.

Hit06 was found in the RNA Polymerase-Associated Protein (RAP94), a 94 kDa viral polypeptide that associates with DNA-dependent RNA polymerase molecules, which is believed to confer specificity to the RNA polymerase for promoters of early genes through its association with VETF [33]. The amino acid sequence of RAP94 associated with Hit06 was LVIFPTHLKIEIER. Both the EMBOSS PatMatMotif tool querying the PROSITE database and searching RAP94 protein against the PROSITE database failed to find matches.

**Figure 7.**
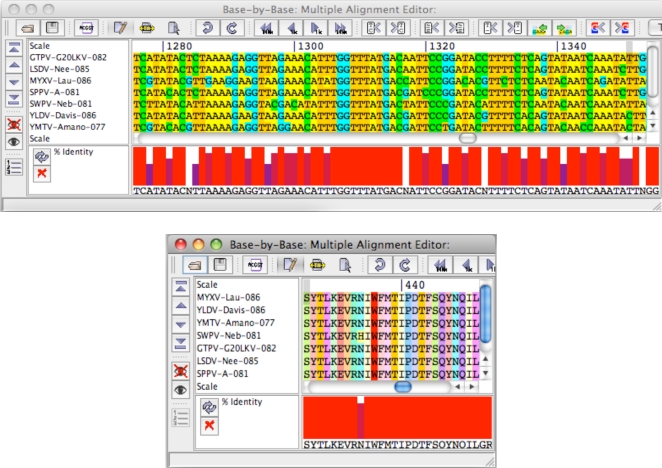
DNA and protein alignments of a super-conserved region in the VETF gene (m081R).

The fact that no hits were found in the PROSITE database does not dispute the fact that these sequences are still unusually well conserved. Sinc,e the PROSITE database looks at all proteins belonging to the same protein groupings, the minimal motifs in the PROSITE database represent the minimal commonalities between proteins from a wide range of hosts, thus it is likely that these matches have poxvirus-specific functions. This analysis therefore does not refute the possibility of conserved functions in these sequences that remain to be identified. To further support the hypothesis these hits have conserved functions, DNA and protein alignments of corresponding regions in the Morphogenesis/Viral Early Transcription Factor gene, *m081R* were examined (Figure 7)

**Figure 8.**
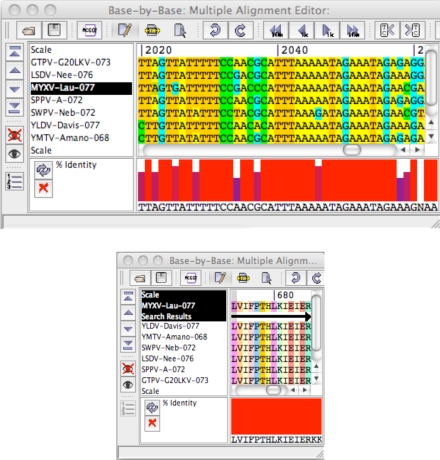
DNA and protein alignments of Hit06.

These results demonstrated that conservation at the protein level did not necessitate high conservation at the DNA level. However, upon examining protein and DNA alignments of Hits 05 and 06, conservation was observed at both the protein and DNA sequence level, further supporting the idea that these coding region hits are unusually well conserved (Figure 8).

#### 3.5.2. Codon Degeneracy

**Figure 9.**
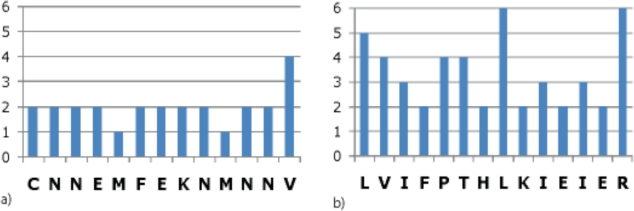
Histograms showing the degeneracy of each amino acid in the protein sequences corresponding to **(a)** Hit05 and **(b)** Hit06.

Although amino acid sequences may be conserved to maintain important protein functions, given codon degeneracy, one would not expect the encoding DNA sequences to be as well conserved as found for Hit05 and Hit06. However, a region of amino acids encoded by codons with low degeneracy rather than a series of amino acids with four- or six-fold codon degeneracy might explain the DNA conservation. To test this possibility, histograms were made showing the number of degenerate codons encoding the amino acid in each position of the protein sequences for Hit05 (Figure 9a) and Hit06 (Figure 9b), based on the genetic code.

**Figure 10.**
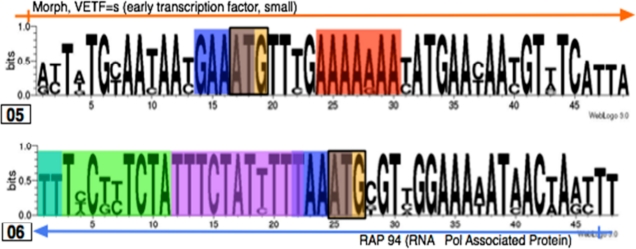
Hit05 and 06 logos with promoter annotations.

The results show that Hit05 is indeed made up of amino acids with relatively low codon degeneracy, including two methionine residues; this may explain the high conservation of this region. However, this does not seem to be the situation for Hit06, which is made up of amino acids with two- to six-fold codon degeneracy. Thus, the DNA conservation is not explained by amino acid conservation with low degeneracy codons. Interestingly, Hits 05 and 06 contain motifs that closely resemble poxvirus promoter elements, even with respect to their overlap with ATG (Met) codons (Figure 10).

The generation of truncated proteins has not been previously observed in poxviruses [34]. Similarly, the dogma of one gene–one protein has not been questioned in poxviruses because the viruses replicate in the cytoplasm in the absence of splicing. However, evolution of viruses has generated many methods by which the protein complement of a virus is modified [35–38] and the generation of novel secondary promoters within established coding regions of poxvirus genes would expand the protein repertoire of the virus by allowing the production of truncated proteins or novel proteins if alternative reading frames were used.

### 4. Conclusions

The discovery of the conserved sequence element (CSE) raised the question of whether or not a 42 nt sequence that is perfectly or near-perfectly conserved in seven different poxviruses from four different genera is unusual. In this study, we have discovered, using the JaPaFi program, that there are in fact a significant number of comparable sequences in this set of genomes. Thus, the CSE is actually part of a larger conserved sequence that is only one of 11 hit sequences that are unusually well conserved among these genomes. Nine of 11 of these hits contain conserved promoter elements, as summarized below, and we hypothesized that the presence of particularly well-conserved promoters partially accounted for most of the conservation observed in these hits. We developed a scoring method based on position scores that represent the degree of conservation at each position of a sequence alignment, as observed in alignment Logos, and we found that the conservation scores for the hits were significantly higher than those obtained for a control set of 10 different promoters.

The 11 hits were analyzed for conserved functions using a number of sequence-based bioinformatics methods, including promoter element searches based on the consensus sequences of the three classes of poxvirus promoters and motif searches within the hits themselves. We found that Hit01 contains the CSE, which was later shown to act as a promoter in poxviruses. Hit01 also includes 10 nts upstream of the beginning of the CSE, which is likely explained by the fact that these JaPaFi searches allowed more differences between the matching regions. The flanking region around the CSE is believed to be part of the promoter for a cytoplasmic protein gene (MYXV m018L), for which translation initiates downstream of the hit. Hits 02, 04, 07 and 10 all contain bidirectional promoters, where promoter elements for two divergently transcribed, opposite strand genes overlap in the non-coding sequence between the genes. In the cases of Hits 04 and 07, these bidirectional promoters make up almost the entire hit, which may explain the conservation of these hits, since the promoter elements constrain the nucleotide makeup of these regions. Hits 02 and 10, however, contain lengthy stretches flanking the bidirectional promoters that are also highly conserved, suggesting that these regions may have conserved functions in addition to the bidirectional promoters. Hits 03, 08 and 09 also contain promoters, which may account for parts of the conservation observed in these hits; however, the majority of each of these hits falls within the coding sequence of nearby genes. This results in two constraints on these sequences; protein sequence conservation as a result of the conservation of these genes, and DNA sequence conservation due to conserved promoter elements. Hits 05 and 06 fell entirely within coding sequences although motifs resembling poxvirus promoters were identified in both. These motifs raised the question of whether or not protein products were produced from transcripts initiating at these alternate start sites. The hit sequences also raised the question of why conservation was observed at the DNA level when it is not required in order for the protein sequence to be conserved. The codon degeneracy of the nucleotides in these protein sequences showed that Hit05 is mostly made up of codons with two-fold degeneracy, limiting the possible variation at the DNA level to a degree that might partially explain the conservation of the hit. Hit06, however, consists mostly of codons with three- to six-fold degeneracy. Since its protein sequence can be encoded by a greater variety of DNA sequences, its high level of conservation suggests that there might be another, non-coding, function associated with this DNA sequence. The protein sequences of neither of these hits were found to be a part of known conserved protein domains when queried against the Prosite database. Therefore, the high degree of conservation observed at the DNA level remains unexplained, and other novel conserved functions may yet exist in the DNA. A single promoter element was identified in Hit11, acting on the DNA Processivity Factor gene. However, this only accounted for part of the sequence, suggesting that the remaining portion may have an unknown conserved function.

Searches identified a motif surrounding the translation starts sites of late genes. This motif brings together the highly conserved TAAAT motif that makes up the initiator site of late promoters and the Kozak sequence, suggesting that it is functional in the DNA as a promoter and mRNA to promote translation.

Although consensus sequences exist for poxvirus promoters, these sequences allow for a great deal of variation since, by nature, the promoters themselves vary in sequence. Therefore it should be noted that although the presence of conserved promoter elements has been considered a suitable explanation for the conservation of the regions of the hits in which these promoter elements fall, they are unusually well conserved even for poxvirus promoters.

### Future Work

Since this analysis has been conducted entirely on the set of seven poxvirus genomes used in Brunetti’s analysis [9], a logical next step would be to investigate more genomes. Applying JaPaFi to a set consisting of one model species from each genus in the poxvirus family may be better suited to identify regions with functions that are conserved within the whole family. It should be noted that sets of genomes to be analyzed together should have similar GC content since too much variation in base composition will likely influence results.

Similar analyses can be conducted on different virus families to identify sequences that may have very different family-specific conserved functions. Coronaviruses, for instance, contain a ribosomal signal in the genome sequence between two ORF. This signal forms an RNA pseudoknot, causing a frame shift that enables the translation of a different ORF. Applying JaPaFi to various sets of viral genomes might identify other DNA or RNA sequences with conserved functions.

## Figures and Tables

**Table 1.** The seven genomes from the poxvirus subfamily *Chordopoxvirinae* used in this study.

**Genus**	**Species**	**GenBank Accession**	**Abbreviation**
Capripoxvirus	Goatpox virus strain G20-LKV	AY077836	GTPV
Capripoxvirus	Lumpy skin disease virus strain Neethling 2490	NC_003027	LSDV
Leporipoxvirus	Myxoma virus strain Lausanne	NC_001132	MYXV
Capripoxvirus	Sheeppox virus strain A	AY077833	SPPV
Suipoxvirus	Swinepox virus strain Nebraska 17077-99	NC_003389	SWPV
Yatapoxvirus	Yaba-like disease virus strain Davis	NC_005179	YLDV
Yatapoxvirus	Yaba monkey tumor virus strain Amano	NC_002632	YMTV

**Table 2.** Hit counts for varying lengths and allowed differences, as observed by running JaPaFi and Longest Common Substring on a set of genomes consisting of GTPV, LSDV, MYXV, SPPV, SWPV, YLDV and YMTV (see Table 1 for abbreviations). Length (*S*) is on the vertical axis, number of differences (*K*) on the horizontal.

*S \ K*	**0**	**1**	**2**	**3**	**4**	**5**	**6**	**7**
**15**	16	303						
**16**	12	115						
**17**	11	57						
**18**	10	31	417					
**19**	9	27	189					
**20**	6	21	117					
**21**	5	15	70	423				
**22**	4	15	55	250				
**23**	3	13	47	177				
**24**	2	11	28	111				
**25**	2	11	25	98				
**26**	1	10	22	83				
**27**	1	8	15	50	148	464		
**28**	1	7	15	45	130	358		
**29**	1	5	13	37		284		
**30**	1	4	9	24	76	188		
**31**	1	4	6	24	65			
**32**	1	3	6	20	60	148		
**33**	0	3	5	14	34			
**34**	0	3	5	12	30	93		
**35**	0	3	4	10	27		184	
**36**	0	3	4	9	22	61		
**37**	0	3	4	8	19		115	
**38**	0	3	4	8	14	43		
**39**	0	2	4	4	11		80	
**40**	0	2	4	3	10	28		
**41**	0	2	3	3	9	26		
**42**	0	1	3	3	6	16	47	
**43**	0	1	3	3	6	14	38	
**44**	0	1	3	3	6	12	35	
**45**	0	1	2	3	4	6	26	
**46**	0	1	2	3	3	5	25	
**47**	0	1	2	3	3	5	23	
**48**	0	1	2	3	3	5	18	
**49**	0	1	2	3	3	5	14	
**50**	0	1	2	3	3	4	12	
**51**	0	0	2	3	3	3	5	
**52**	0	0	2	3	3	3	5	18
**53**	0	0	1	3	3	3	5	18
**54**	0	0	1	3	3	3	4	11
**55**	0	0	1	2	3	3	3	9
**56**	0	0	1	1	2	3	3	8
**57**	0	0	1	1	2	3	3	5
**58**	0	0	0	1	2	3	3	5
**59**	0	0	0	1	1	3	3	5
**60**	0	0	0	1	1	3	3	5
**61**	0	0	0	0	1	2	3	3
**62**	0	0	0	0	1	2	3	3
**63**	0	0	0	0	1	2	3	3
**64**	0	0	0	0	1	2	3	3
**65**	0	0	0	0	1	2	3	3
**66**	0	0	0	0	1	2	3	3
**67**	0	0	0	0	0	2	3	3
**68**	0	0	0	0	0	2	3	3
**69**	0	0	0	0	0	2	2	3
**70**	0	0	0	0	0	2	2	3

**Table 3.** The final set of highly conserved sequences (hits) and the positions within the *Myxoma virus* genome.

**Final set of hits**			
**hit**	**start**	**stop**	**length**
01	18521	18573	53
02	52335	52414	80
03	54146	54199	54
04	66601	66645	45
05	80165	80212	48
06	68525	68573	49
07	100085	100138	54
08	102112	102203	92
09	102281	102321	41
10	104268	104341	74
11	106243	106299	57

**Table 4.** Conservation scores calculated for **(a)** hits and **(b)** baseline sequences. Section **(c)** compares averages for the hits *versus* those for the baseline sequences.

**a)**	**Hit**	**Total Info (bits)**

	Hit01	85.93
	Hit02	144.02
	Hit03	90.43
	Hit04	81.75
	Hit05	82.28
	Hit06	83.35
	Hit07	90.27
	Hit08	165.36
	Hit09	73.69
	Hit10	125.23
	Hit11	95.45
**b)**		
	**Ortholog Group**	**Total Info (bits)**

	Baseline1	47.19
	Baseline2	70.13
	Baseline3	83.94
	Baseline4	49.38
	Baseline5	77.80
	Baseline6	53.79
	Baseline7	50.02
	Baseline8	71.27
	Baseline9	52.13
	Baseline10	60.46
**c)**		
		**Total Info (bits)**

	**t**	3.91
	**Std. Deviation**	10.227
	**Degrees of Freedom**	19
	**p**	0.0009
